# Deciphering the Heterogeneity of Pancreatic Cancer: DNA Methylation-Based Cell Type Deconvolution Unveils Distinct Subgroups and Immune Landscapes

**DOI:** 10.3390/epigenomes9030034

**Published:** 2025-09-05

**Authors:** Barbara Mitsuyasu Barbosa, Alexandre Todorovic Fabro, Roberto da Silva Gomes, Claudia Aparecida Rainho

**Affiliations:** 1 Department of Genetics, Microbiology and Immunology, Institute of Biosciences, São Paulo State University (UNESP), Botucatu 18618-680, SP, Brazil; barbara.mitsuyasu@unesp.br; 2 Graduate Program in Internal Medicine, Ribeirão Preto Medical School, University of São Paulo (USP), Ribeirão Preto 14049-900, SP, Brazil; alexandretodofabro@gmail.com; 3 Department of Pharmaceutical Sciences, North Dakota State University, Fargo, ND 58103, USA; roberto.gomes@ndsu.edu

**Keywords:** immune microenvironment, tumor purity, DNA methylation age, gene modules, regulatory networks

## Abstract

**Background:** Pancreatic ductal adenocarcinoma (PDAC) is a highly heterogeneous malignancy, characterized by low tumor cellularity, a dense stromal response, and intricate cellular and molecular interactions within the tumor microenvironment (TME). Although bulk omics technologies have enhanced our understanding of the molecular landscape of PDAC, the specific contributions of non-malignant immune and stromal components to tumor progression and therapeutic response remain poorly understood. **Methods:** We explored genome-wide DNA methylation and transcriptomic data from the Cancer Genome Atlas Pancreatic Adenocarcinoma cohort (TCGA-PAAD) to profile the immune composition of the TME and uncover gene co-expression networks. Bioinformatic analyses included DNA methylation profiling followed by hierarchical deconvolution, epigenetic age estimation, and a weighted gene co-expression network analysis (WGCNA). **Results:** The unsupervised clustering of methylation profiles identified two major tumor groups, with Group 2 (n = 98) exhibiting higher tumor purity and a greater frequency of *KRAS* mutations compared to Group 1 (n = 87) (*p* < 0.0001). The hierarchical deconvolution of DNA methylation data revealed three distinct TME subtypes, termed hypo-inflamed (immune-deserted), myeloid-enriched, and lymphoid-enriched (notably T-cell predominant). These immune clusters were further supported by co-expression modules identified via WGCNA, which were enriched in immune regulatory and signaling pathways. **Conclusions:** This integrative epigenomic–transcriptomic analysis offers a robust framework for stratifying PDAC patients based on the tumor immune microenvironment (TIME), providing valuable insights for biomarker discovery and the development of precision immunotherapies.

## 1. Introduction

The tumor microenvironment (TME) is a complex ecosystem comprising malignant epithelial cells, stromal elements, infiltrating immune cells, and an extensive extracellular matrix. Interactions among these components regulate key aspects of tumor biology, including proliferation, invasion, and metastasis [1,2]. While transcriptomic profiling has been used to characterize TME heterogeneity, recent advances have positioned cell-type deconvolution of DNA methylation data as a robust method to infer immune and stromal cell abundances in bulk tumor tissues due to its stability and cell lineage specificity [3]. Furthermore, genomic and transcriptomic analyses have revealed distinct TME signatures across different cancer types, underscoring the tissue-specific nature of TME interactions and their implications for cancer progression and therapeutic response [4,5,6,7,8]. For instance, stromal cells, such as cancer-associated fibroblasts (CAFs), can modulate TME composition and communicate with immune cells to influence tumor development. Studies have shown that CAFs not only promote but also inhibit tumors in addition to the heterogeneity of the TME [9,10]. The immune microenvironment is a critical component of the TME and influences both antitumor immunity and immune evasion mechanisms. Immunotherapies have demonstrated remarkable efficacy in certain types of cancers by harnessing the host immune response against malignant cells [11,12]. TME heterogeneity is also influenced by the diverse nature of driver mutations within tumors, which are responsible for initiating and maintaining neoplastic growth [13,14].

Among solid tumors, pancreatic ductal adenocarcinoma (PDAC) is particularly distinguished by its dense stromal architecture and unique patterns of immune infiltration, both of which contribute to its dismal prognosis and resistance to therapy. As a result, pancreatic cancer represents a major public health challenge. Difficulties in early detection, the lack of specific signs or symptoms, the absence of effective screening strategies, and the inherently aggressive behavior of the disease are associated with the advanced staging at diagnosis and resistance to available treatment modalities, leading to therapeutic failure and unfavorable clinical outcomes [15].

Morphological and molecular intra- and inter-tumor heterogeneity in pancreatic cancer are closely associated with disease progression. Histologically, this malignancy is characterized by a pronounced desmoplastic reaction, featuring dense fibrotic stroma and low tumor cellularity (5–20% cancer cells), which poses a significant challenge for the interpretation of molecular data obtained from tumor biopsies. The desmoplastic profile within the TME of pancreatic cancer could also pose a problem for drug delivery [16,17]. Although the pancreatic TME usually has an immunosuppressive profile, it varies considerably across different tumor subtypes and undergoes dynamic changes during disease progression [18]. Using an in vivo model, Li et al. investigated cell-intrinsic factors contributing to tumor immune heterogeneity and sensitivity to immunotherapy. By co-injecting pancreatic cancer cell clones into immunocompetent mice, the authors identified distinct tumor immune microenvironments (TIMEs), recapitulating both T cell-inflamed and non-T cell-inflamed phenotypes. Moreover, they observed that the non-T cell-inflamed phenotype was dominant and that intra-tumoral CD8^+^ T cells played a critical role in determining the response to immunotherapy [12].

The integration of multi-omics profiling data provided by the Cancer Genome Atlas (TCGA) enables a comprehensive understanding of tumor biology by linking genomic, epigenomic, transcriptomic, and clinical features across diverse cancer types. It should be highlighted that analysis of the mutational profile revealed that *KRAS* oncogene is mutated in approximately 93% of samples, with a subset of pancreatic tumors showing multiple variants in this gene, in addition to evidence of biallelic mutations [16]. Also, 60% of cancers negative for *KRAS* mutation had a mutation in the RAS-MAPK signaling pathway gene, such as *RREB1* (ras responsive element binding protein 1) [16,19]. Gain-of-function variants of *KRAS* occur in the early stages of pancreatic cancer, before its progression to invasive carcinoma [20,21]. Beyond its well-established oncogenic functions, emerging evidence suggests that pathogenic *KRAS* variants also contribute to shaping the immunosuppressive phenotype of the tumor microenvironment including lung and pancreatic cancers [22]. Recent DNA methylation profiling has identified distinct subgroups within lung adenocarcinoma, which are associated with specific oncogenic drivers (*KRAS* and *TP53* mutations) and with varying compositions of immune cell populations [23].

In recent years, novel computational methodologies have been used to infer the complex composition of tumors into their constituent cellular elements. These methods deconvolve tumor samples based on various types of genomic information, such as microarray [24], transcriptomic [25,26], and methylation data [27]. In this study, we reanalyzed publicly available DNA methylation data from TCGA to evaluate the heterogeneity of pancreatic cancer. To gain new insights into the relationship between the pancreatic cancer methylome and the tumor immune microenvironment (TIME), we first assessed the influence of tumor sample purity on DNA methylation-based clustering and epigenetic age. The hierarchical deconvolution of the TIME was then conducted to predict immune and stromal cell-type composition, following PAM (partitioning around medoids) clustering, which was used to classify tumor samples based on estimated tumor cell fractions. Finally, we examined TIME patterns in relation to the expression of specific gene modules, co-regulated networks, and histomorphological parameters.

## 2. Results

### 2.1. Global DNA Methylation Profiling Identifies Distinct Patterns in Pancreatic Cancer

**Figure 1:**
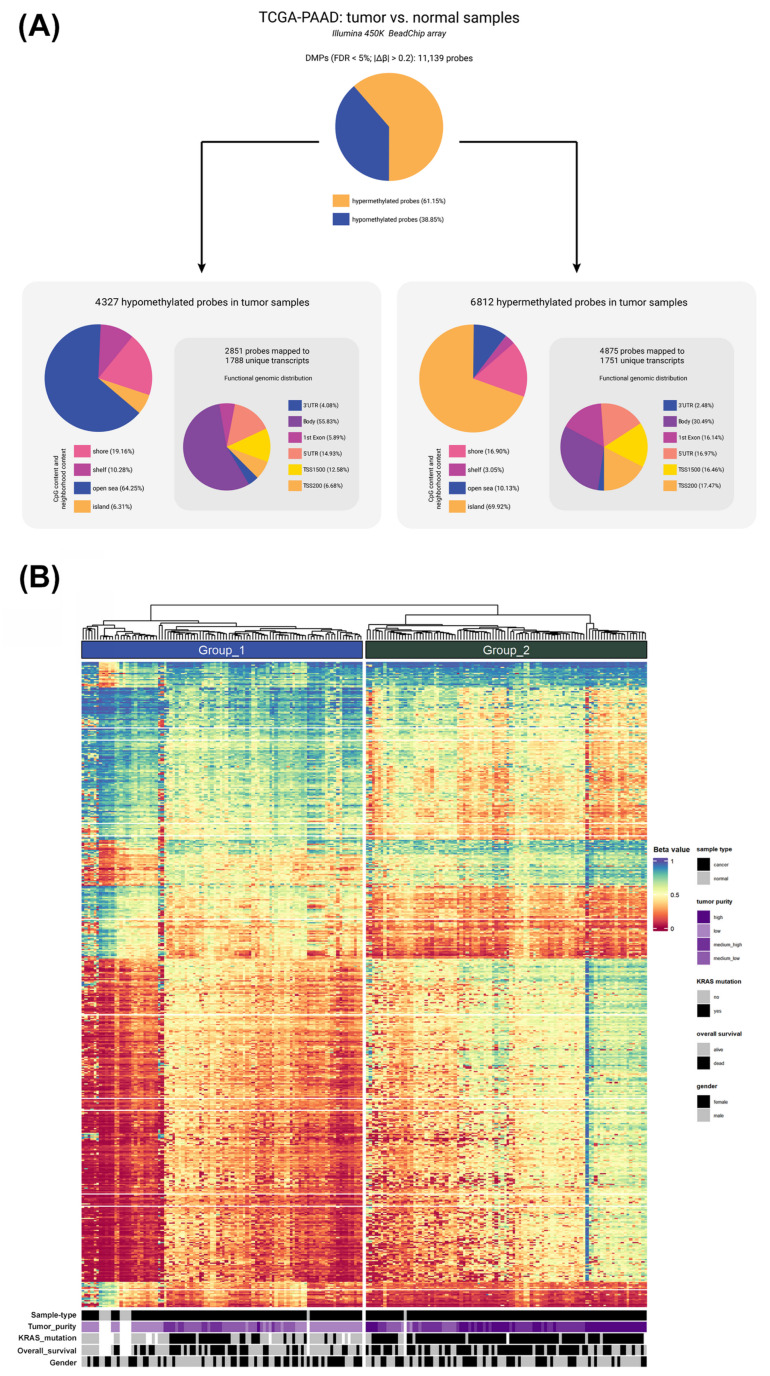
Differentially methylated positions (DMPs) in pancreatic cancer between tumor and normal samples (FDR < 5%; |∆β| > 0.2). (**A**) Frequency distribution of DMPs according to their CpG content and neighborhood context (shore, shelf, open sea, and island) and functional genomic distribution (3’UTR, body, 1st exon, 5’UTR, TSS1500, and TSS200). (**B**) Heatmap of DNA methylation profile of DMPs. Samples’ clinicopathological and molecular features are shown at the bottom.

Differential methylation analysis revealed distinct methylation patterns in pancreatic cancer compared with their normal counterparts, identifying 11,139 differentially methylated positions (DMPs). These positions were associated with 3328 unique gene coordinates obtained from the UCSC Genome Browser (UCSC Genes track, hg38 assembly). Figure 1A shows the distribution of hypo- and hypermethylated positions according to CpG content, neighborhood context, and functional genome distribution. Hypomethylated positions (n = 4327) were concentrated in the open sea (64.25%) and gene body (55.83%) regions, whereas hypermethylated positions (n = 6812) were mapped to the CpG islands (69.92%) and promoter regions (33.93%) (Supplementary Table S1).

The unsupervised hierarchical clustering of DMPs identified two major groups with distinct molecular and clinical characteristics. Group 1 included 87 tumor samples and 9 normal samples, whereas Group 2 consisted predominantly of tumor samples, with only one normal sample among a total of 96. These distinct methylation profiles were associated with specific tumor features. Notably, Group 1 was enriched for low/medium–low purity, whereas Group 2 was predominantly composed of medium–high/high purity samples, showing a highly significant difference between groups (*p* < 0.0001) (Table 1). In addition, Group 2 exhibited a significantly higher frequency of *KRAS* mutations compared to Group 1 (*p* < 0.0001) (Figure 1B). *KRAS* mutation status was also associated with positive epigenetic age acceleration (*p* = 0.0128; Supplementary Figure S1). Furthermore, the overall survival outcomes differed significantly between the clusters, and Group 2 had a lower survival rate (*p* = 0.0046) (Figure 1B; Table 1).

### 2.2. Hierarchical DNA Methylation-Based Tumor Deconvolution Reveals Distinct Patterns of Tumor Immune Microenvironment in Pancreatic Cancer

**Figure 2:**
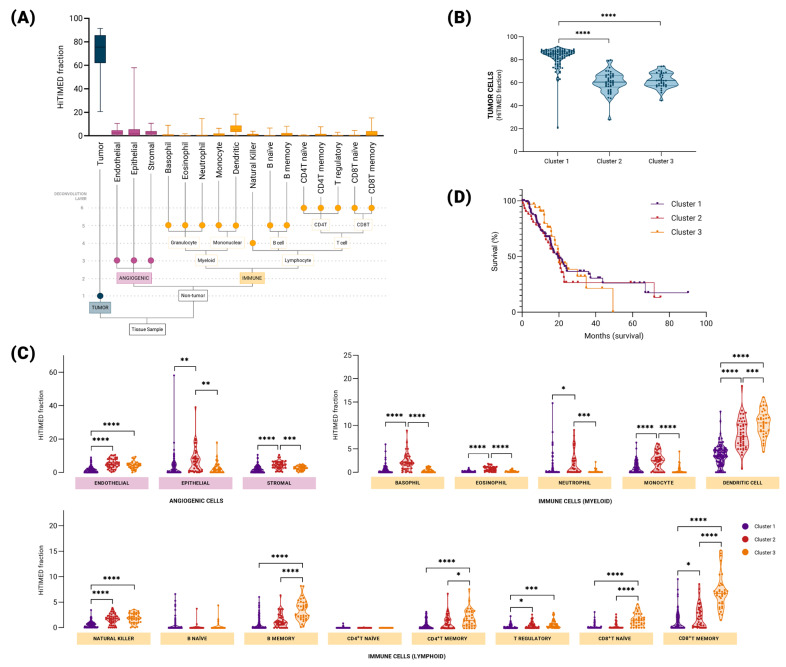
DNA methylation-based tumor deconvolution. (**A**) Distribution of cell composition in TCGA-PAAD tumor samples (n = 178). The structure of the hierarchical model used to deconvolve pancreatic cancer tumor samples is illustrated below the X axis, showing the six hierarchical layers represented in the graph. (**B**) Tumor cell distribution of the three clusters. Clusters were distinguished using HiTIMED cell fractions PAM clustering. (**C**) Comparison of non-tumor cell populations in PAM clustering-derived groups of TCGA-PAAD. ANOVA with Tukey’s post hoc multiple comparisons test was employed to assess the differences between clusters (* *p* ≤ 0.05; ** *p* ≤ 0.01; *** *p* ≤ 0.001; **** *p* ≤ 0.0001). (**D**) Overall survival of pancreatic cancer patients stratified according to PAM-clusters (Mantel–Cox test; *p*-value 0.7749).

Figure 2A shows the heterogeneous patterns of the estimated cell population composition across 178 TCGA-PAAD tumor samples. The hierarchical distribution of the cell-type fractions is shown in Figure 2B (Supplementary Table S2a). PAM clustering distinguishes three sets characterized by distinct cellular compositions. Cluster 1 contained a higher proportion of tumor cells (Figure 2B). Although the proportion of tumor cells was comparable between clusters 2 and 3, the composition of non-tumor cells differed (Figure 2B,C). Our analysis revealed the distinct distributions of angiogenic components across the identified clusters. Specifically, cluster 2 exhibited higher levels of epithelial and stromal cells than clusters 1 and 3 did. Conversely, cluster 1 displayed an elevated proportion of endothelial cells compared with clusters 2 and 3. Regarding immune components, we identified the distinct enrichment of myeloid and lymphoid cell populations within PAM clusters. Cluster 2 exhibited higher levels of basophils, eosinophils, neutrophils, monocytes, and dendritic cells than clusters 1 and 3 did within the myeloid cell subset. Additionally, the proportion of dendritic cells differed between clusters 1 and 3. Regarding lymphoid cells, cluster 1 displayed lower levels of natural killer and T regulatory cells than clusters 2 and 3. Furthermore, cluster 3 demonstrated elevated proportions of B memory, CD4^+^T memory, and CD8^+^T naïve cells compared to clusters 1 and 2. CD8^+^T memory cells exhibited a similar pattern to dendritic cells, with cluster 3 having higher levels than clusters 1 and 2, while cluster 2 also displayed higher levels than cluster 1. Notably, no significant differences were observed between the three clusters in the naïve B and CD4^+^T naïve cell populations (Figure 2C). Thus, the three identified PAM clusters were named according to their proportions of immune-cell fractions. Cluster 1, characterized by a lower abundance of non-tumoral cells, was designated hypo-inflamed (immune-deserted). Cluster 2 displayed a predominance of myeloid cells and was labeled as myeloid-enriched. Cluster 3, enriched with lymphoid cells, notably T cells, was named as lymphoid-enriched. Interestingly, no difference (*p* = 0.7749) was observed in overall survival when comparing the three PAM clusters, despite their different cell composition profiles (Figure 2D).

A high concordance rate was observed between the predicted PAM clusters and the histomorphological analysis. The lymphoid-enriched group was characterized by the presence of lymphoid aggregates, whereas the myeloid-enriched samples exhibited histiocytic and macrophagic infiltrates. Extensive desmoplasia was confirmed as the main feature of hypo-inflamed samples (Supplementary Figure S2 and Supplementary Table S2b). To further support these findings, the expression levels of a panel of immune cell marker genes were compared across the clusters. The lymphoid cell marker genes *CD3D*, *CD3E*, *CD247*, *CD8A*, and *KLRD1* showed significantly increased expression in the lymphoid-enriched group compared with the hypo-inflamed and myeloid-enriched groups (Supplementary Figure S3).

### 2.3. Weighted Correlation Network Analysis (WGCNA) Uncovers Group of Genes Associated with Immune Groups in Pancreatic Cancer

**Figure 3:**
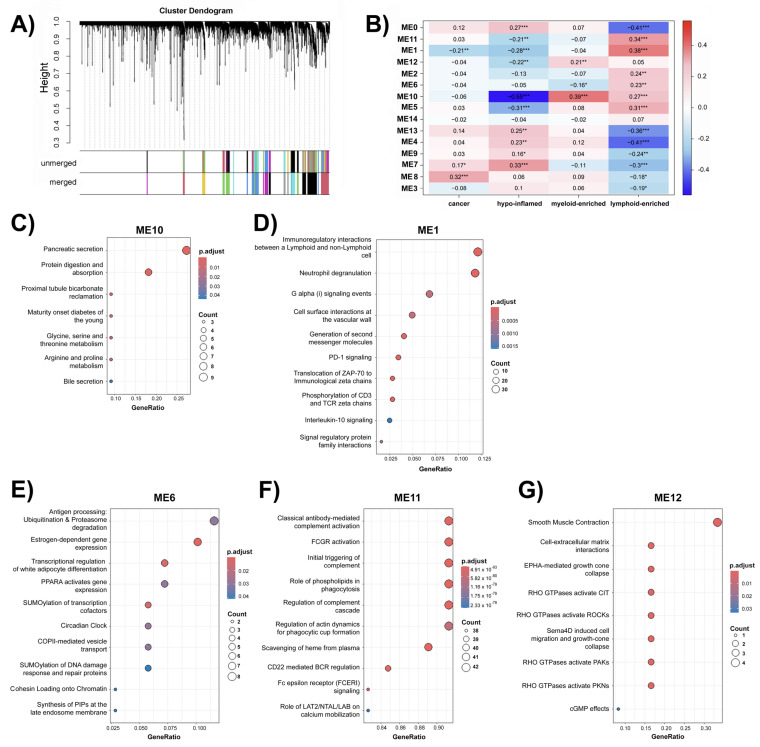
Visual representation of weighted gene correlation network analysis (WGCNA) obtained from the TCGA-PAAD RNA-seq dataset. (**A**) Cluster dendrogram based on topological overlap distances in gene expression patterns of primary solid tumors and adjacent normal samples associated with pancreatic cancer. Each color displayed in the bar below the dendrogram represents a module, which signifies a cluster of genes. Genes displayed in white indicate a lack of similar expression patterns resulting in their exclusion from any specific module. Clusters within the unmerged bar represent the diverse patterns of expression initially identified. Unmerged clusters were combined according to their similarity, resulting in the final clusters represented in the merged bar. (**B**) Correlation analysis of module eigengenes with traits-disease status (cancer vs. normal samples) or immune tumor groups (hypo-inflamed, myeloid-enriched, and lymphoid-enriched), with the correlation values between module eigengenes and traits shown numerically within the cells.The color gradient of the cells corresponds to the correlation values, as illustrated by the scale bar on the right. Asterisks within the cells signify the level of statistical significance (* *p* ≤ 0.05; ** *p* ≤ 0.01; *** *p* ≤ 0.001). (**C**) Kyoto Encyclopedia of Genes and Genomes (KEGG) enrichment analysis for module 10. (**D**–**G**) Reactome pathway enrichment analysis of ME1, ME6, ME11, and ME12 genes, respectively.

In our analysis of the TCGA-PAAD RNA-seq dataset, WGCNA revealed the presence of 14 modules representing consensus clusters (Figure 3A). The correlation between each module and immune group is shown in Figure 3B. Subsequent enrichment analyses were conducted to elucidate the biological pathways and functions associated with these co-expressed gene modules. Notably, our investigation identified module (ME) 10 as pivotal for the three predicted immune phenotypes, showing a negative correlation with the immune-deserted cluster and a positive correlation with immune-enriched clusters. Furthermore, KEGG (Kyoto Encyclopedia of Genes and Genomes) enrichment analysis revealed that the network comprising genes closely associated with pancreatic function (Figure 3C).

Genes in ME1 were negatively correlated with hypo-inflamed clusters and positively correlated with the lymphoid-enriched clusters. ME6 was negatively correlated with myeloid enrichment and positively correlated with lymphoid-enriched clusters. Similarly, ME11 exhibited a negative correlation with hypo-inflamed and a positive correlation with lymphoid-enrichment, whereas ME12 demonstrated a negative correlation with hypo-inflamed samples and a positive correlation with myeloid-enrichment (Figure 3B).

Reactome enrichment analysis revealed the distinct biological processes and pathways associated with each network. ME1 was enriched in processes related to immunoregulatory pathways (Figure 3D). ME6 and ME11 were enriched in the signaling and regulatory pathways (Figure 3E,F). Finally, ME12 was enriched in processes related to cellular signaling and cytoskeletal regulation pathways (Figure 3G).

## 3. Discussion

Unraveling intratumor heterogeneity at both molecular and cellular levels is essential for advancing our understanding of pancreatic cancer biology, particularly regarding how complex interactions influence clinical outcomes and responses to therapy in this disease defined by an immunosuppressive TME. The molecular and histological landscapes of the PDAC vary widely within individual tumors, and the diverse cell populations present in the TME influence prognosis and clinical outcomes [18,28]. At the molecular level, distinct tumor cell subpopulations can be identified within a single tumor because of myriad factors, such as clonal evolution and acquired genetic and epigenetic alterations [29,30]. Furthermore, tumor tissues also comprise non-tumor cells, including stromal, vascular, and immune components, thus conferring cellular heterogeneity [18]. The heterogeneity of epithelial cancer cells observed in precursor lesions of pancreatic cancer intensifies as the disease advances toward invasive carcinoma [31]. Additionally, there are non-tumoral cell populations, including immune cells, that also displayed intra- and inter-tumor heterogeneity distributions in pancreatic cancer [32]. Here, we adopted a multidimensional methodology to explore tumor heterogeneity using DNA methylation profiling, DNA methylation age, RNA-seq, and mutational data to characterize and classify tumor samples based on their predicted TME composition. Distinct tumor microenvironments with distinct immune profiles have also been identified. In addition, we observed an association between *KRAS* pathogenic variants and positive epigenetic age acceleration.

We first employed traditional bulk analysis techniques, including genomic, transcriptomic, and epigenomic approaches. The unsupervised clustering of differentially methylated positions (DMPs) revealed two distinct DNA methylation profiles. We observed a significant association between tumor purity and methylation profiles, with lower-purity tumors exhibiting distinct patterns compared to higher-purity tumors. These distinct profiles in low-purity samples are consistent with admixture and likely reflect the contribution of non-tumor cell populations, particularly stromal and immune cells, which dilute or mask tumor-specific methylation signals. Also, the group with the highest tumor purity also exhibited a higher frequency of *KRAS* pathogenic variants, which are driver mutations commonly associated with pancreatic cancer. This association suggests a potential link between tumor purity and the detection of *KRAS* mutations. It is plausible that, in samples with lower purity, the abundance of non-tumor cells may dilute the presence of tumor-derived epithelial cells, potentially impacting the detection sensitivity for *KRAS* mutations. Consequently, the observed enrichment of *KRAS* mutations in samples with higher tumor purity may reflect a greater proportion of tumor cells capable of harboring this driver mutation. However, traditional bulk methods do not consider diversity within the TME. The advent of single-cell and spatial analysis technologies has enabled the characterization of distinct cell populations and their spatial organization within tumors [28,29]. Moreover, these technologies have facilitated the identification of gene expression patterns within specific cell populations associated with treatment responses in pancreatic cancer [30]. Integrative approaches offer valuable insights into the prognostic significance of TIME profiles in PDAC and hold promise for advancing our understanding of the tumor microenvironment and its influence on pancreatic cancer outcomes and therapeutic response [31,32,33]. However, univariate survival analysis showed no significant association between the three TIME-predicted subtypes and overall survival. This lack of difference may reflect the influence of additional factors in overall survival. A recent study [34] reported that genes correlated with T cell enrichment patterns exhibited subtype-specific prognostic effects. Multivariable analysis indicated improvements in median overall survival for patients with high methylation levels of *TGFB2*, *IFI27*, and *TGFB3*, whereas high *TGFB1* methylation was associated with shorter median survival. These findings highlight how gene-specific methylation patterns and immune contexture can differentially influence survival, underscoring the importance of integrating molecular and clinical variables in future analyses. Another study [35] combined immunophenotyping, stromal scoring, and histomorphological profiling in a cohort of 112 PDAC cases, including 25 long-term survivors. While no distinct mutational differences were observed between short- and long-term survivors, variations in TIME composition were evident. Notably, macrophage infiltration correlated with poorer overall survival, whereas long-term survivors showed increased CD4^+^ T cell infiltration and predominantly inactive stromal profiles [35].

*KRAS* driver mutations have been implicated in modulating the tumor epigenome, particularly by inducing focal changes in DNA methylation patterns rather than widespread global alterations. These effects are likely context-dependent and mediated through downstream signaling pathways that regulate epigenetic modifiers. A comprehensive analysis involving 47 cell lines underscored the significant impact of tumor cell type on DNA methylation profiling, overshadowing the influence of *KRAS* driver mutations. However, investigations into 11 pancreatic cancer isogenic cell line pairs revealed that *KRAS* knockdown induced cell line-specific alterations in DNA methylation, suggesting a role for *KRAS* in mediating epigenetic regulation [36]. In a recent study, lung cancer samples were categorized into hot or cold immune phenotypes using deconvolution results obtained with MethylCIBERSORT. Consistent with our results, a lower DNA methylation age was also correlated with the presence of driver gene mutations in lung cancer [23].

Our findings using the cell-type deconvolution of DNA methylation data also revealed distinct subtypes of pancreatic cancer, and we were able to identify three clusters with distinct TIME compositions. These methylation patterns revealed heterogeneous cellular compositions that reflected the tumor microenvironment. We observed pancreatic tumors with low immune infiltration (hypo-inflamed) and tumors exhibiting different immune infiltrates (myeloid-enriched and lymphoid-enriched) characterized by distinct immune lineage cell populations. Remarkably, the cellular subtypes predicted by deconvolution closely align with the characterization of the tumor immune microenvironment using cell type-specific markers and multiplex immunohistochemical imaging [37]. This analysis highlights the inherent heterogeneity of immune profiles within pancreatic tumors, which is laborious and time-consuming to capture fully through routine clinical assessment using histopathological approaches. The deconvolution approach provides a powerful, high-throughput alternative for directly profiling the complex immune landscape of these tumors.

Weighted correlation network analysis (WGCNA) uncovered distinct gene modules associated with immune subtypes in pancreatic cancer, revealing the underlying tumor-immune interactions. Of particular significance are ME1, ME6, ME10, ME11, and ME12, which exhibit distinct gene expression patterns that correlate with their respective immune profiles. Remarkably, genes within ME10 were associated with all the three immune groups predicted in our study. KEGG enrichment analysis revealed their relevance in pancreatic function (Figure 3C). This observation supports the validity of our analysis, underscoring its appropriateness given the utilization of pancreatic cancer samples.

Cluster 2 was enriched with myeloid lineage cells (myeloid-enriched), which are the main hematopoietic cells in the human body. The reactome pathways enriched in module 12 may be involved in the cytoskeletal rearrangements necessary for myeloid cell migration and phagocytosis. For instance, cell-extracellular matrix (ECM) interactions are crucial for myeloid cells, such as macrophages and neutrophils. These interactions influence the migration, adhesion, and pro-inflammatory activities of myeloid cells within the tumor by remodeling the ECM [38]. The RHO family of small GTPases is a key regulator of actin cytoskeleton in myeloid cells. Macrophages can promote angiogenesis within tumors by secreting factors, such as Sema4D and VEGF, which activate RHO GTPases [39,40]. Activation of these RHO GTPases and their downstream effectors, such as ROCKs (Rho-associated protein kinases), PAK (p21-activated kinases), and PKNs (protein kinases N), triggers cytoskeletal rearrangements that drive myeloid cell migration and infiltration into the tumor [41,42].

Cluster 3 displayed a predominance of lymphoid cells (lymphoid-enriched), notably CD4^+^T memory, CD8^+^T-naïve, and CD8^+^T memory cells (Figure 2C). Upon activation by antigens, naïve T cells become effector cells that target and destroy cancer cells as a part of the immediate immune response. After tumor cells are cleared, memory T cells remain vigilant within the immune system, ready to proliferate, and respond more efficiently if the same antigen reappears [43]. The enrichment terms in modules were positively correlated with the lymphoid-enriched cluster (ME1, ME6, and ME11), as immunoregulatory interactions between lymphoid and non-lymphoid cells are crucial for the activation and function of T cells, which interact with antigen-presenting cells [43]. Processes such as the translocation of ZAP-70 (Zeta-chain-associated protein kinase 70) to the immunological synapse and subsequent phosphorylation of CD3 and TCR zeta chains are key events in T cell activation and signaling pathways [44]. The PD-1 signaling pathway, represented by programmed cell death protein 1 (PD-1), is a fundamental inhibitory receptor universally expressed by activated T-cells [45,46]. Pathways essential for antigen processing and presentation alongside CD22-mediated B-cell receptor (BCR) regulation are pivotal for adaptive immunity mediated by lymphoid lineage cells [47,48]. Additionally, classical antibody-mediated complement activation, the initial triggering of complement, and the regulation of the complement cascade support the action of effector T cells by facilitating tumor cell destruction and promoting inflammation to recruit additional immune cells [49].

Together, the complementary approaches applied in our study provide converging evidence that specific co-regulated gene modules capture immune-related variation and may underlie functional differences among the predicted TIME subtypes. Nonetheless, we acknowledge the limitations inherent to findings derived and tested within the same cohort. Therefore, further investigations in larger independent cohorts are required to confirm the robustness of these results. In addition, future validation in both experimental models and well-annotated clinical datasets will be essential to reinforce the translational relevance of our observations and to support their potential application in clinical practice.

## 4. Materials and Methods

**Figure 4:**
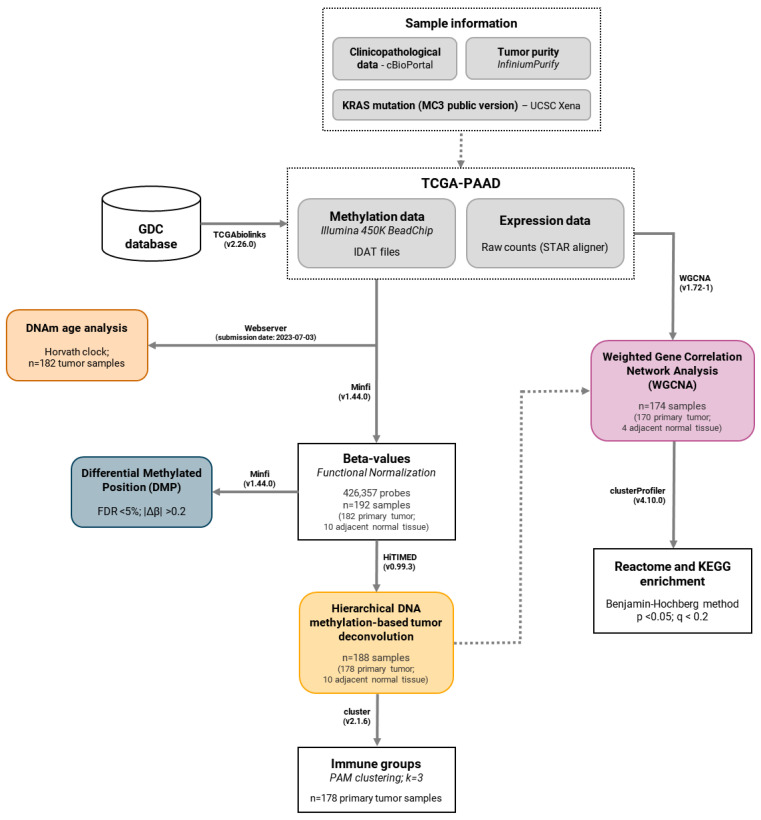
Flowchart summarizing the study design and the methodology employed for the identification of tumor-infiltrating immune cells from methylation data and the construction of a weighted gene correlation network from RNA-Seq in pancreatic cancer.

A flowchart summarizing the study design and methodology employed for the identification of tumor-infiltrating immune cells from DNA methylation data and the construction of a weighted gene correlation network from RNA-Seq is shown in Figure 4.

### 4.1. DNA Methylation Profiling in Pancreatic Cancer

#### 4.1.1. Data Acquisition and Pre-Processing

DNA methylation data from the TCGA Pancreatic Cancer (TCGA-PAAD) cohort, sourced from the Cancer Genome Atlas Research Network (https://www.cancer.gov/tcga; accessed on 20 February 2024) (RRID:SCR_003193), were selected for this study. Raw intensity data (IDAT) files were retrieved from the Genomic Data Commons (GDC) database with TCGA biolinks R package (v.2.26.0) [50]. Data were preprocessed using the Minfi Bioconductor R package (v.1.44.0) (RRID:SCR_012830) [51] and functional normalization, an unsupervised approach that leverages control probes as surrogates for unwanted variation, was applied as recommended [52]. Following functional normalization, one tumor sample was excluded because it failed to pass the quality control (QC) parameters (probes with low quality, probes mapped to X/Y chromosomes, cross-reactive probes, and those overlapping positions were removed). Subsequently, cross-reactive probes that could potentially hybridize to multiple genomic locations owing to sequence similarity [53], probes located on the X or Y chromosome, and polymorphic CpGs that overlapped Single Nucleotide Variants (SNVs) with a minor allele frequency (MAF < 0.05) based on dbSNP 137 using the annotation file for the 450 K array at the University of California at Santa Cruz (UCSC) hg19 assembly (GRCh37 build reference sequence) [54] were filtered. In addition, probes with poor-quality signals (detection *p* < 0.05) and low bead counts (<3) in >5% of the samples were removed. DNA methylation values, described as beta values, were calculated for 192 tissue samples, including 182 pancreatic tumor samples and 10 normal samples.

Clinicopathological information was retrieved from the cBioPortal (https://www.cbioportal.org/, accessed on 26 July 2024) (RRID:SCR_014555). The MC3 public-version mutation data [55] for *KRAS* mutations in tumor samples were obtained from the UCSC Xena repository (https://xena.ucsc.edu/, accessed on 26 July 2024). The tumor purity of TCGA-PAAD samples was estimated using the InfiniumPurify package (v.1.3.1) [56].

#### 4.1.2. Differentially Methylated Positions Analysis

Differentially methylated CpG positions (DMPs) between tumor and normal samples were detected using the Minfi R package (v.1.44.0) [51]. Probes meeting the criteria of a False Discovery Rate (FDR) of less than 5% and |∆β| > 0.2 were considered statistically significant. The significant probes were annotated using a manifest file [57]. The probes resulting from the DMP analysis were annotated to UCSC Reference Sequencing (RefSeq) genes (RRID:SCR_003496) using the annotation file for the 450 K array in the UCSC hg19 assembly [54]. DMPs were categorized based on their CpG content, neighborhood context (shore, shelf, open sea, and island), and functional genomic distribution (3′-UTR, body, 1st exon, 5′-UTR, TSS1500, and TSS200).

### 4.2. DNA Methylation Age Analysis

DNA methylation (DNAm) age was determined using Horvath clock, a multi-tissue algorithm that employs a weighted average of methylation levels at specific CpG sites throughout the genome to predict epigenetic age directly from IDAT files [58]. The epigenetic age acceleration (EAA) was subsequently defined based on the residuals obtained by regressing the DNAm age on chronological age. A positive EAA (>0) indicates an acceleration of epigenetic aging, whereas a negative EAA (<0) indicates a deceleration relative to chronological aging (Supplementary Figure S1a).

### 4.3. Hierarchical DNA Methylation-Based Tumor Deconvolution

The proportion of cell types in each sample was determined using HiTIMED R package (v.0.99.3) [59]. This analysis utilized beta values from the TCGA-PAAD dataset using a six-layer-tumor-type-specific model. A total of 17 cell types representing the three major components of the tumor microenvironment were identified: tumor, angiogenic (endothelial, epithelial, and stromal), and immune (basophil, eosinophil, neutrophil, monocyte, dendritic, natural killer (NK), B-naïve, B-memory, CD4^+^T-naïve, CD4^+^T memory, T regulatory, CD8^+^T-naïve, and CD8^+^T memory) cells [59]. The cluster R package (RRID:SCR_013505) [60] was used for partitioning around medoid (PAM) clustering to identify TIME patterns based on the obtained specific cell fractions.

### 4.4. Gene Expression Analysis Using a Network-Based Approach

#### 4.4.1. Data Acquisition and Pre-Processing

The TCGA-PAAD gene expression quantification cohort was obtained from the Genomic Data Commons (GDC) database (RRID:SCR_014514) using TCGAbiolinks R package (v2.26.0) [50] as raw counts aligned to the human genome using the STAR RNA-seq aligner (RRID:SCR_004463) [61]. The dataset comprised 182 samples (178 primary solid tumors and four adjacent normal tissues) and 60,660 distinct transcripts. Normalized expression levels of selected genes were retrieved directly from UCSC Xena repository (https://xena.ucsc.edu/, accessed on 26 July 2024).

The data were preprocessed according to weighted correlation network analysis (WGCNA) recommendations [62]. Initially, low-count genes (<15) in > 75% of samples were excluded. Subsequently, the raw counts were normalized by the median ratio method followed by variance-stabilizing transformations (VST) using the DESeq2 R package (v1.41.10) (RRID:SCR_015687) [63]. Quality control (QC), employing the goodSamplesGenes function from the WGCNA R package (v1.72-1) [61], deemed all samples and genes acceptable. Outlier samples were identified using two distinct methodologies: hierarchical clustering and principal component analysis (PCA) and removed.

#### 4.4.2. Weighted Gene Correlation Network Analysis (WGCNA)

The WGCNA R package (v.1.72-1) (RRID:SCR_003302) [62] blockwise modules function was used to construct a weighted co-expression network, where genes were represented as nodes and the connectivity between them was established based on pairwise correlations derived from their expression profiles [64]. To obtain a scale-free topology, a power of 14 was chosen as the soft threshold for creating an adjacency matrix. Genes were grouped into different modules according to their similar expression patterns using a topological overlap matrix (TOM) of dissimilarity measures and the branches of a dendrogram derived from the average linkage hierarchical clustering. The identified modules were then correlated with sample traits (sample type and deconvolution-derived immune groups) using Pearson’s correlation coefficients. Key driver genes in the modules of interest were identified using intramodular connectivity.

#### 4.4.3. Enrichment Analysis of WGCNA Modules

Entrez gene identifiers (Entrez ID) for the UCSC RefSeq genes of each module were retrieved using the biomaRt R package (v2.58.0) [65,66] using an annotation database [67]. Kyoto Encyclopedia of Genes and Genomes (KEGG) (RRID:SCR_012773) and Reactome (RRID:SCR_003485) pathway enrichment analyses were performed for selected WGCNA modules using the ClusterProfiler R package (v.4.10.0) (RRID:SCR_016884) [68]. KEGG and Reactome enrichment were analyzed using the Benjamini–Hochberg (BH) method to adjust the *p*-values (*p*-value < 0.05; *q*-value < 0.2).

### 4.5. Histopathological Analysis

Histopathological evaluation was performed using slides from the Cancer Digital Slide Archive (http://cancer.digitalslidearchive.net, accessed on 15 August 2025) [69], which integrates pathology imaging with clinical and omics data from TCGA project. Manually curated annotations of lymphocytic aggregates, eosinophils, histiocytes, macrophages, and desmoplasia were assessed in hematoxylin and eosin (H&E)-stained sections and compared across the three predicted TIME patterns. The semiquantitative analysis of lymphoid aggregates considered the number and density (≤2 aggregates were scored as 1 when loosely organized and 2 when densely compacted, whereas cases with >2 aggregates were scored as 3 and 4, when loosely organized or densely compacted, respectively). Histiocytic infiltrates were classified according to their density as well as their peri or intra tumoral localization. Sixty tissue samples were included, comprising twenty randomly selected cases per predicted TIME group.

### 4.6. Statistical Analysis

The EAA was calculated based on the residuals obtained through linear regression of DNAm age on chronological age. Fisher’s exact test was used to assess the association between DNA methylation-based groups and the clinical and molecular characteristics of pancreatic cancer. ANOVA with Tukey’s post hoc multiple comparisons test was employed to assess the differences in HiTIMED-estimated cell subpopulations among PAM clustering-derived groups. The overall survival of PAM clusters was calculated using the Mantel–Cox test. Statistical analysis was performed using the GraphPad Prism software (v10.2.1) (RRID:SCR_002798) (* *p* ≤ 0.05, ** *p* ≤ 0.01, *** *p* ≤ 0.001, **** *p* ≤ 0.0001).

## 5. Conclusions

In summary, this study reveals distinct TIME subtypes in pancreatic cancer based on DNA methylation patterns, reflecting underlying immune heterogeneity and aligning with prior histological classifications. The integration of methylation-based deconvolution and gene co-expression analysis supports a robust framework for patient stratification and highlights potential immune-related biomarkers and therapeutic epi-targets. These findings offer new avenues for improving prognostic accuracy and treatment strategies in pancreatic cancer.

## Figures and Tables

**Table 1:** Clinical and molecular distribution of TCGA-PAAD data used in DNA methylation profiling using 182 tumor samples.

		Group 1		Group 2		
Variables	Samples Total	N	(%)	N	(%)	*p*-Value *
Gender	182	87		95	(100%)	0.6553
Female		41		41		
Male		46		54	(100%)	
Age (years)		87		95		0.6362
<50		11		9		
>50		76		86		
Overall Survival	182	87	(100%)	95	(100%)	0.0046
Alive		50	(57.5%)	34	(35.8%)	
Dead		37	(42.5%)	61	(64.2%)	
KRAS mutation	173	80	(100%)	93	(100%)	<0.0001
Presence		30	(37.5%)	84	(90.3%)	
Absence		50	(62.5%)	9	(9.7%)	
Tumor purity	182	87	(100%)	95	(100%)	<0.0001
Low		46	(52.9%)	2	(2.1%)	
Medium–low		27	(31.0%)	12	(12.6%)	
Medium–high		13	(14.9%)	37	(38.9%)	
High		1	(1.1%)	44	(46.3%)	

* Fisher’s exact test.

## Data Availability

The results published here are based on data generated by the TCGA Research Network (https://www.cancer.gov/tcga). All data generated or analyzed during this study are included in this published article and its Supplementary Files.

## Supplementary Materials

The following supporting information can be downloaded at: https://www.mdpi.com/article/10.3390/epigenomes9030034/s1, Supplementary Figures: Supplementary Figure S1: A. Epigenetic age acceleration (EAA) is defined based on the residuals from the linear regression of DNAm age on chronological age. B. *KRAS* mutation status and EAA exhibit a significant relationship (Fisher’s exact test; *p* = 0.0128); Supplementary Figure S2: Representative tissue slides of TIME patterns; Supplementary Figure S3: Distribution of expression levels of selected genes encoding lymphoid cell markers across the predicted TIME groups. Table S1: Supplementary Table S1a: List of 4327 hypomethylated probes); Supplementary Table S1b: List of 6812 hypermethylated probes, (FDR < 5%; ∆β > 0.2 and ∆β < −0.2, respectively) in tumor samples of the Cancer Genome Atlas (TCGA) Pancreatic Cancer (PAAD) cohort. Table S2: Supplementary Table S2a. DNA methylation-based tumor deconvolution of cell types in the Cancer Genome Atlas (TCGA) Pancreatic Cancer (PAAD) cohort. Supplementary Table S2b. Comparison between HiTIMED-predicted groups and histopathological evaluation of slides from the Cancer Digital Slide Archive (http://cancer.digitalslidearchive.net, accessed on 15 August 2025) for a subset of TCGA-PAAD cohort.

## Abbreviations

The following abbreviations are used in this manuscript:

BCR	B-cell receptor
CAFs	Cancer-associated fibroblasts
CD22	Cluster of Differentiation 22
CD247	CD247 molecule
CD3	Cluster of differentiation 3
CD4^+^T	Cluster of differentiation 4 T lymphocyte
CD3D	CD3 delta subunit of T-cell receptor complex
CD3E	CD3 epsilon subunit of T-cell receptor complex
CD3G	CD3 gamma subunit of T-cell receptor complex
CD8A	CD8 subunit alpha
CD8^+^T	Cluster of differentiation cytotoxic T lymphocytes
CpG	Cytosine Guanine dinucleotide
DMP	Differentially methylated position
DNAm	DNA methylation
EAA	Epigenetic Age Acceleration
ECM	Extracellular matrix
FDR	False discovery rate
GDC	Genomic Data Commons
GTPase	Guanosine triphosphatases
HiTIMED	Hierarchical Tumor Immune Microenvironment Epigenetic Deconvolution
IFI27	Interferon alpha inducible protein 27
IDAT	Illumina Data (raw intensity data)
KLRD1	Killer cell lectin-like receptor D1
KEGG	Kyoto Encyclopedia of Genes and Genomes
*KRAS*	Kirsten rat sarcoma viral oncogene
MS4A1	Membrane spanning 4-domains A1
MC3	Multi-Center Mutation Calling in Multiple Cancers
ME	Module Eigengene
NCAM1	Neural cell adhesion molecule 1
PAAD	Pancreatic adenocarcinoma
PAKs	p21-activated kinase
PAM	Partitioning around medoids
PD-1	Programmed cell death protein 1
PDAC	Pancreatic ductal adenocarcinoma
PKNs	Protein kinases N
RAS-MAPK	Ras-mitogen-activated protein kinase
RHO	Ras homolog
ROCKs	Rho-associated protein kinases
*RREB1*	Ras-responsive element binding protein 1
RRID	Research Resource Identifier
Sema4D	Semaphorin 4D
SNP	Single Nucleotide Polymorphism
TCGA-PAAD	The Cancer Genome Atlas—Pancreatic adenocarcinoma cohort
TCR	T cell receptor
TGFB1	Transforming growth factor beta 1
TGFB2	Transforming growth factor beta 2
TGFB3	Transforming growth factor beta 3
TIME	Tumor immune microenvironment
TME	Tumor microenvironment
TOM	Topological overlap matrix
*TP53*	Tumor suppressor p53 gene
TSS	Transcription Start Site
UCSC	The University of California, Santa Cruz
UTR	Untranslated region
VEGF	Vascular Endothelial Growth Factor
WGCNA	Weighted Gene Co-expression Network Analysis
ZAP-70	Zeta-chain-associated protein kinase 70
