# Supplementary material for: Deciphering the Heterogeneity of Pancreatic Cancer: DNA Methylation-Based Cell Type Deconvolution Unveils Distinct Subgroups and Immune Landscapes
Source: Epigenomes. 2025 Sep 5;9(3):34. doi: 10.3390/epigenomes9030034 (PMC12452622; doi:10.3390/epigenomes9030034)
Supplement: Supplementary file 1 [file epigenomes-09-00034-s001.zip › Supplementary Figures.pdf]

Supplementary Figures

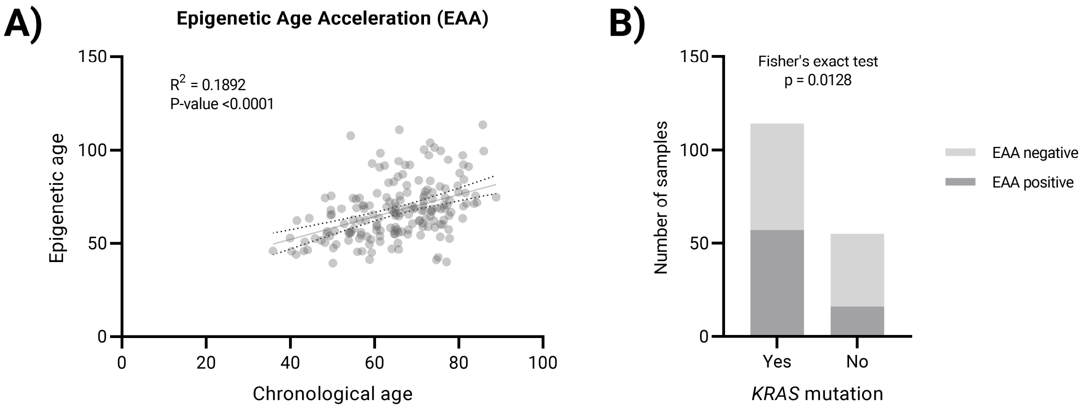

Supplementary Figure S1. (A) Epigenetic age acceleration (EAA) is defined based on the residuals from the linear regression of DNAm age on chronological age. (**B)** *KRAS* mutation status and EAA exhibit a significant relationship (Fisher's exact test; *p*=0.0128).

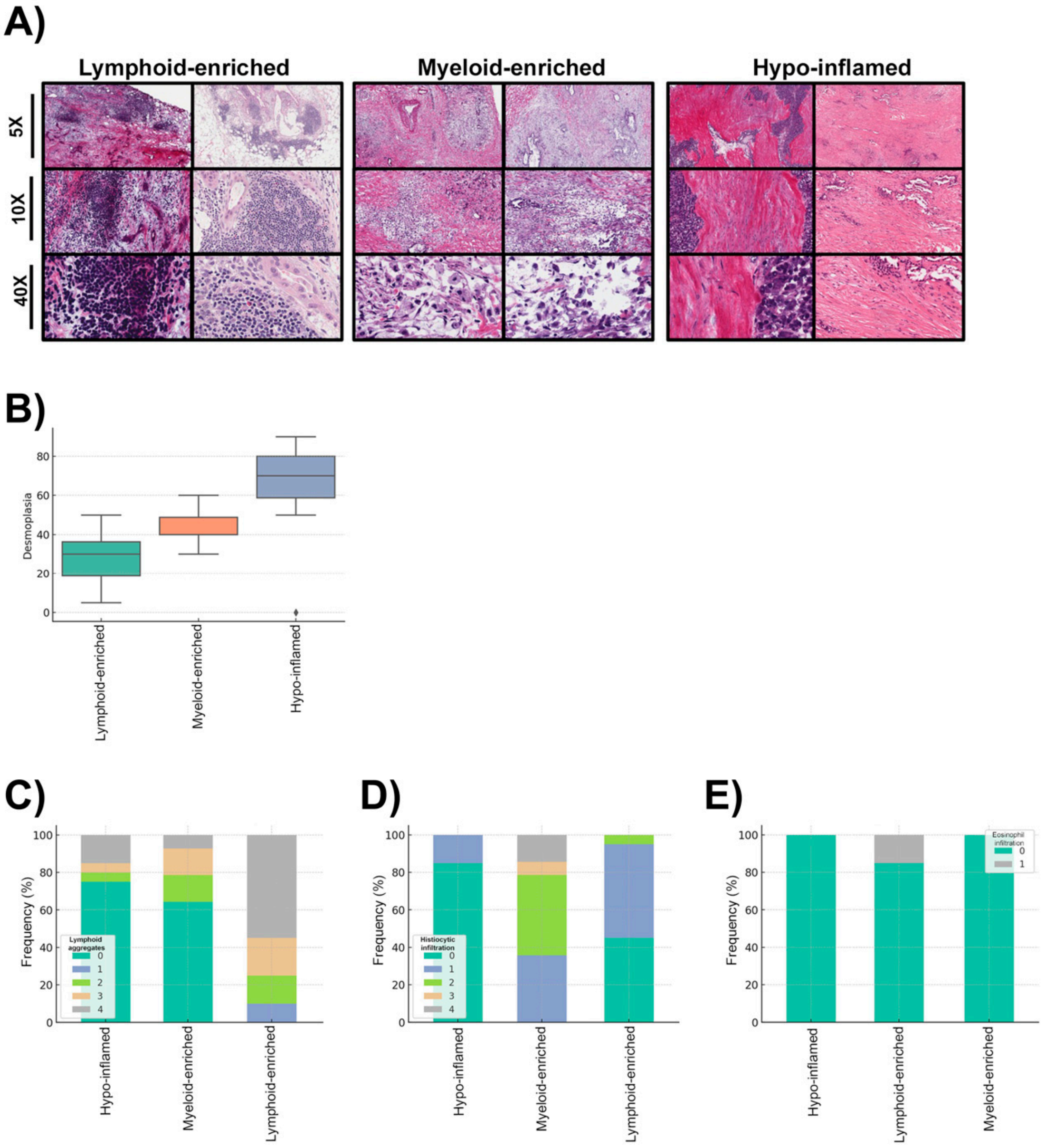

Supplementary Figure S2. Representative tissue slides of TIME patterns. (**A**) Histomorphological panel of lymphoid-enriched, myeloid-enriched, and hypo-inflamed groups at 5X, 10X, and 40X magnifications. The lymphoid group is characterized by the presence of lymphoid aggregates, while the myeloid group shows histiocytic and macrophagic infiltrates. The hypo-inflamed group displays extensive desmoplasia, sometimes hyalinized, without lymphoid or macrophagic aggregates. Hematoxylin and eosin staining. (**B**) Box-and-whisker plots of desmoplasia across groups, showing a graded increase from lymphoid-enriched to myeloid-enriched and highest levels in the hypo-inflamed group ($p<0.00001$, Kruskal-Wallis's test). (**C**) Stacked bar chart depicting the distribution of semi-quantitative scores for lymphoid aggregates concentrated in lymphoid-enriched group ($p<0.001$) and (**D**) higher grades of histiocytic infiltration are concentrated in the myeloid-enriched group ($p<0.000001$), while lymphoid-enriched and hypo-inflamed groups are largely low/absent. (**E**) Eosinophil infiltration indicating low frequency across groups with only occasional positivity.

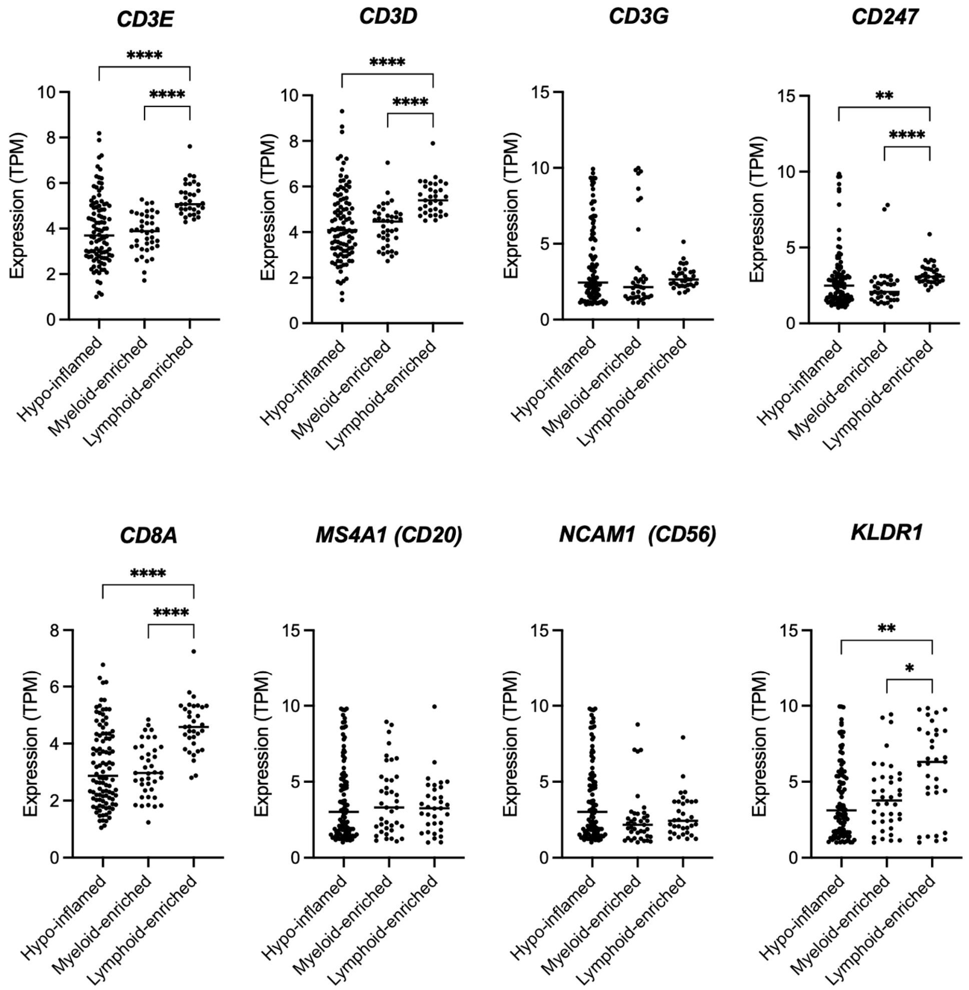

Supplementary Figure S3. Distribution of expression levels of selected genes encoding lymphoid cell markers across the predicted TIME groups. (*p≤0.05; **p≤0.01; ****p≤0.0001). (TPM = Transcripts Per Million).
